# An Open-Source Mouse Chronic EEG Array System with High-Density MXene-Based Skull Surface Electrodes

**DOI:** 10.1523/ENEURO.0512-22.2023

**Published:** 2024-02-09

**Authors:** Li Ding, Aashvi Patel, Sneha Shankar, Nicolette Driscoll, Chengwen Zhou, Tonia S. Rex, Flavia Vitale, Martin J. Gallagher

**Affiliations:** ^1^Department of Neurology, Vanderbilt University School of Medicine, Nashville 37232, Tennessee; ^2^Departments of Bioengineering and Neurology, Center for Neuroengineering & Therapeutics, University of Pennsylvania, Philadelphia 19104, Pennsylvania; ^3^Department of Ophthalmology & Visual Sciences, Vanderbilt University School of Medicine, Nashville 37232, Tennessee; ^4^Center for Neurotrauma, Neurodegeneration, and Restoration, Corporal Michael J. Crescenz Veterans Affairs Medical Center, Philadelphia 19104, Pennsylvania; ^5^Department of Veteran’s Affairs, Tennessee Valley Health System, Nashville 37212, Tennessee

**Keywords:** computer-assisted, electrodes, electrophysiology, epilepsy, GABAA, mild traumatic brain injury, receptors, signal processing

## Abstract

Electroencephalography (EEG) is an indispensable tool in epilepsy, sleep, and behavioral research. In rodents, EEG recordings are typically performed with metal electrodes that traverse the skull into the epidural space. In addition to requiring major surgery, intracranial EEG is difficult to perform for more than a few electrodes, is time-intensive, and confounds experiments studying traumatic brain injury. Here, we describe an open-source cost-effective refinement of this technique for chronic mouse EEG recording. Our alternative two-channel (EEG2) and sixteen-channel high-density EEG (HdEEG) arrays use electrodes made of the novel, flexible 2D nanomaterial titanium carbide (Ti_3_C_2_T*_x_*) MXene. The MXene electrodes are placed on the surface of the intact skull and establish an electrical connection without conductive gel or paste. Fabrication and implantation times of MXene EEG electrodes are significantly shorter than the standard approach, and recorded resting baseline and epileptiform EEG waveforms are similar to those obtained with traditional epidural electrodes. Applying HdEEG to a mild traumatic brain injury (mTBI) model in mice of both sexes revealed that mTBI significantly increased spike–wave discharge (SWD) preictal network connectivity with frequencies of interest in the β-spectral band (12–30 Hz). These findings indicate that the fabrication of MXene electrode arrays is a cost-effective, efficient technology for multichannel EEG recording in mice that obviates the need for skull-penetrating surgery. Moreover, increased preictal β-frequency network connectivity may contribute to the development of early post-mTBI SWDs.

## Significance Statement

Electroencephalography (EEG) is a critical technique used to study neurological activity in rodents. Commonly used EEG procedures require time-consuming skull-penetrating surgeries that may confound the experiments. Here we provide a cost-effective solution for obtaining two-channel (EEG2) and high-density EEG (HdEEG) recordings on the skull surface thus avoiding major surgery. We compared this HdEEG system to traditional EEG recordings and then used it to determine the effects of mild traumatic brain injury on preictal network connectivity. This novel open-source EEG system will contribute to the electrophysiological characterization of mouse behaviors and seizure activity.

## Introduction

Electroencephalography (EEG) is an indispensable tool in epilepsy, sleep, and behavioral research. The most common role of EEG in rodent experiments is to quantify seizures and stage sleep (del Campo et al., 2009) and thus only needs to record signals from a limited number of brain regions (often two channels, EEG2). However, experiments using high-density EEG (HdEEG, 16–32 channels) are necessary for experiments designed to localize neurophysiological activity within the rodent brain with high resolution and accuracy.

Human EEG electrodes are metal cups filled with an electrolyte gel that establishes electrical contact with the scalp and must be repeatedly replaced during extended recordings due to gel drying (Searle and Kirkup, 2000). In addition to electrolyte drying, gel-filled cup electrodes cannot be used for chronic rodent EEG recordings due to the difficulty in attaching them to the scalp/skull and the propensity of the electrolyte to bridge adjacent electrodes on their small skulls. Therefore, current rodent EEG studies typically use 0.1–1.0 mm diameter metal electrodes inserted via cranial burr holes into the epidural space to establish electrode/electrolyte contact and to secure them for chronic recording. Drilling cranial burr holes is a major surgery that could potentially alter physiology. Skull-penetrating surgeries are difficult in young rodents with thin skulls, and they confound experiments studying mild traumatic brain injuries (mTBI), which are not associated with skull fractures. Epidural HdEEG experiments done with homemade arrays involve laborious fabrication and implantation procedures (Wasilczuk et al., 2016; Ding et al., 2019). While HdEEG experiments can also be performed using commercially available ultrathin 16–32 channel electrode arrays that lie on the skull surface (Choi et al., 2010; Jonak et al., 2018, 2020), they still require the placement of three epidural anchoring screws and their cost is prohibitive. There is a need for inexpensive, easily fabricated EEG2 and HdEEG arrays that can be implanted without skull injury.

In this open-source EEG2/HdEEG method, investigators design thin flexible printed circuit boards (PCBs) with electrode pads overlying cortical regions of interest. The electrode pads are then functionalized with soft contacts made with titanium carbide (Ti_3_C_2_T*_x_*) MXene, a biocompatible (Dai et al., 2017), flexible 2D nanomaterial that provides electrical contact between the electrode pad and the skull without the need for electrolyte gel or paste (Driscoll et al., 2021). The EEG2/HdEEG arrays are chronically affixed to the skull surface with UV-curable cement without the need for any skull burr holes. We validated this system by measuring normal and epileptiform EEG2 signals obtained with traditional metal epidural electrodes and MXene electrodes and comparing the spatiospectral distribution of preictal and epileptiform spectral density obtained with MXene HdEEG electrodes with those reported previously using epidural HdEEG arrays. Finally, we used MXene HdEEG arrays to determine the acute effects of mTBI on SWDs. Many mTBI patients have long-term neurological symptoms (Nelson et al., 2019), and elucidating the neurophysiological consequences of mTBI in rodents is a necessary step in developing effective mTBI therapies (Liang et al., 2022).

## Materials and Methods

### Animals

The protocols were approved by the (author university) Animal Care and Use Committee. We used adult mice (20 ± 3 weeks, 26.8 ± 3.8 g) in the DBA/2J background that heterozygously expressed a missense human epilepsy mutation in the GABA_A_ receptor (Gabra1^+/A322D^) that are an established model of epileptiform SWDs (Arain et al., 2012, 2015). Mice had unlimited access to food and water and were housed in a temperature- and humidity-controlled environment with a 12 h lights-on/lights-off cycle with lights on at 0600 (Zeitgeber Time 0, ZT0). After all experiments, mice were killed by CO_2_ asphyxia on the last day of recording and their skulls and brains were examined to evaluate for the presence of visible injury.

### Electrode arrays

We compared MXene electrodes with traditionally used 1.0 and 0.1 mm diameter metal electrodes; 1.0 mm electrodes are more stable than 0.1 mm electrodes while the thinner 0.1 mm electrodes are more suitable for high-density intracranial recordings. The 1.0 mm screw electrodes were commercial EEG2/EMG arrays from Pinnacle (catalog #8201), and the 0.1 mm electrodes were made from thin tungsten rods (Ding et al., 2019).

**Figure 1.**
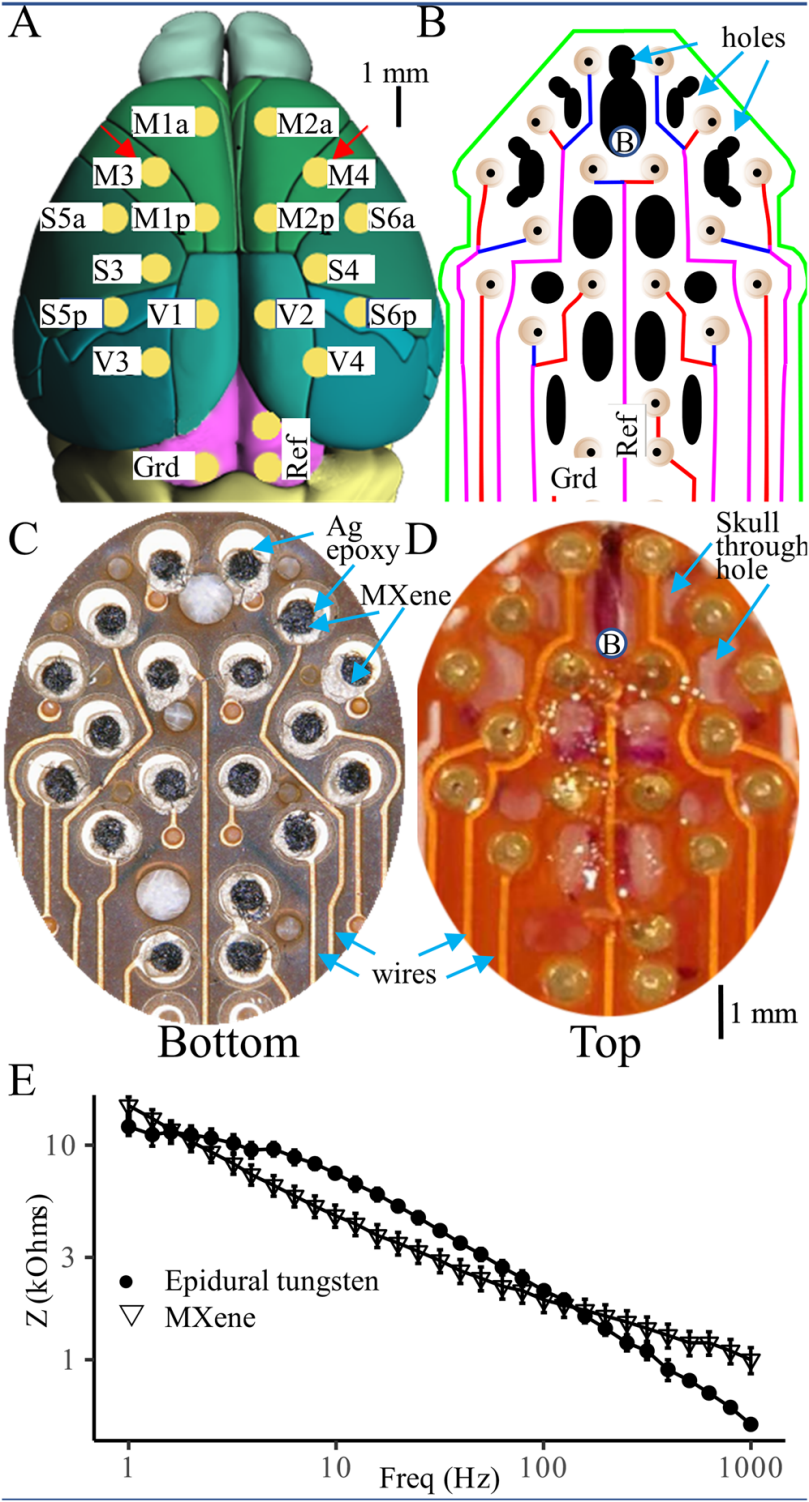
MXene HdEEG fabrication. ***A***, Drawing of the mouse dorsal cortical surface showing the position of the 16 HdEEG channels (yellow) along with the ground (Grd) and reference (Ref) placed over the midbrain. The uppercase M, S, and V letters of the electrode names indicate placement over motor, somatosensory, and visual cortices. The lowercase a and p letters in the M1/2 and S5/6 electrodes indicate anterior and posterior placement. Electrodes M3 and M4 indicated with red arrows are used for EEG2 recordings. The scale bar indicates 1 mm. ***B***, The circuit diagram shows the HdEEG PCB design. Electrode pads (beige) connected with wires (red = top PCB surface, blue = bottom PCB surface, magenta = separate wires on top and bottom surface) that ultimately terminate on connector pads. Black shapes indicate holes where UV-curable cement is placed. The “B” placed at the bottom of the uppermost black hole indicates positioning over bregma that enables reproducible placement; this is used for visual alignment only. ***C***, Photograph of the bottom of a completed MXene HdEEG array. The silver epoxide is silver, MXene is dark gray, and wires are yellow. ***D***, Intraoperative photograph of implanted MXene HdEEG array. Letter “B” indicates bregma. ***E***, Impedance spectra of 0.1 mm epidural (●) and MXene (▽) electrodes measured in PBS solution (*N* = 5).

We used free online software (EasyEDA) to design double-sided flexible PCBs for MXene HdEEG arrays with 16 electrode pads (0.7 mm diameter rings with 0.2 mm holes) placed over the dorsal cortex and ground and reference electrodes placed over the midbrain (Fig. 1*A*,*B*). The coordinates of the electrodes (in mm relative to the bregma positioning hole) were M1a/2a (±0.75, 0.90), M1p/2p (±0.75, −1.30), M3/4 (±1.85, −0.25), S3/4 (±1.95, −2.45), S5a/6a (±2.80, −1.30), S5p/6p (±2.80, −3.45), V1/2 (±0.75, −3.50), V3/4 (±1.80, −4.60), and Grd/Ref (±0.75, −7.00). For the 48 h continuous recordings, EMG electrodes (insulated tungsten wires with exposed tips to be inserted in the nuchal muscles) for the facilitation of sleep staging were connected to the PCB at ±0.80 mm and −8.30 mm. The EEG2 MXene arrays are identical to HdEEG arrays but only use the two recording electrodes over the left and right motor cortex (M3/M4, Fig. 1*A*,*B*). Figure 1, *B* and *C*, shows the electrode locations, the positioning hole in the polyimide array used to visually align it with bregma during the surgery, and the holes through which photocurable cement is applied to affix it to the skull.

The PCBs for the 0.1 mm epidural tungsten EEG2 arrays were identical to the MXene arrays except that the electrode pads were 1.05 mm diameter rings with 0.75 mm holes. Five gold-plated electronic pin receptacle connectors (0.52 mm inner diameter, Mill-Max, 4428-0-43-15-04-14-10-0) were soldered to the electrode pads through the 0.75 mm holes and then strengthened with nonconductive epoxy. The 0.1 mm epidural electrodes were five 3 mm tungsten rods (254 µm diameter, A-M Systems 717200) cut to 3 mm and ground to a 100 µm tip with a Dremel rotary tool and then inserted through the pin receptacle connectors.

For both MXene and tungsten arrays, the wires connecting electrode pads were 150 µm wide. We designed the PCB to have pads for the attachment of either one four-position (EEG2) or two ten-position (HdEEG) rectangular connector sockets (Fig. 1*A*,*B*) to connect the arrays to the EEG amplifier. The use of the four-position EEG2 connector allows the array to interface with a commercial mouse EEG2 acquisition system (Pinnacle). Flexible PCBs using this design were manufactured on 0.2 mm polyimide by a commercial PCB prototyping company (PCBWay).

For the MXene arrays, a polyimide template with holes overlying the electrode pads was made using a CO_2_ laser cutter. Ti_3_C_2_T_x_ MXene aqueous dispersions were provided by Murata Manufacturing. A nonwoven, hydroentangled cellulose polyester (60–40%) blend substrate was infused with the Ti_3_C_2_T*_x_* MXene (20 mg/ml) and thoroughly dried. Circular electrode contacts were then cut out from the MXene-infused substrate using a biopsy punch to form circles (h 0.4 mm, d 500 µm). With the polyimide template placed over the PCB, the circular MXene contacts were applied to each electrode pad using conductive silver epoxy (CircuitWorks CW2400). Rectangular socket connectors were soldered to the PCB connector pads (four-position connector for EEG2 cut from six-position Samtec SFMC-103-T1-*S*-D; two ten-position connectors for HdEEG cut from twenty-position Samtec CLP-110-02-L-D-P-TR).

Samples of both the tungsten electrodes and MXene electrodes were characterized by electrochemical impedance spectroscopy using a Gamry Reference 600 potentiostat. Measurements were conducted at room temperature in 10 mM phosphate-buffered saline (PBS solution, pH 7.4) using a three-electrode configuration, with an Ag/AgCl reference electrode and a graphite rod counter electrode. Impedance spectra were collected at frequencies ranging from 1 Hz to 1 MHz with 10 mV rms AC voltage.

### Surgery

Under isoflurane anesthesia, we made an incision in the dorsal scalp, cleaned and dried the skull, and visually centered the positioning hole over the bregma to ensure reproducible placement. For the epidural EEG2 arrays, the PCBs were attached to the skull with cyanoacrylate adhesive and dental cement. For the epidural recordings, a 29 Ga needle was inserted through the lumen of the electrode connectors to gently drill burr holes in the underlying skull. The 1.0 mm steel screw electrodes or 0.1 mm tungsten electrodes were inserted in the connectors with their tips in the epidural space. The MXene HdEEG arrays were placed on the skull, and a small amount of ultraviolet-curable adhesive (Visbella UF0008CR2P) was applied individually to each access hole while the nearby MXene electrode pads were held firmly against the skull to prevent the epoxy from seeping between the electrode and the skull. A UV light cured the epoxy.

### Overpressure blast mTBI

We used a validated overpressure method for producing mTBI (Heldt et al., 2014; Reiner et al., 2014; Bu et al., 2016; Guley et al., 2016, 2019; Jha et al., 2018; Honig et al., 2021; Mondal et al., 2021). The mice were anesthetized with 2–4% isoflurane in 100% O_2_ throughout the procedure, first in an induction chamber and then via a nasal cannula while the mice were in the overpressure apparatus. After anesthesia induction, the mice were placed in a plastic holding tube, which was then inserted in a second plastic tube that is situated on an *x*–*y* table that can precisely position the skull 4 cm from the barrel of a paintball gun. The dorsum of the mouse skulls (at bregma) was positioned under 6 mm holes in the plastic tubes that align with the barrel and transmit the pressure blast to a precise location in the skull. The pressure of the paintball gun provided a single 40 psi pressure wave with a half-width duration of 7 ms; sham mice experienced the anesthesia and exposure to the apparatus without overpressure. After the sham/mTBI, we measured the righting time, a common immediate assessment of traumatic brain injury (TBI) severity (Kane et al., 2012; Gullotti et al., 2014). Immediately after the mTBI/sham, the mouse was placed on its side and the time until it stood on its paws was considered the righting time.

### EEG recording and analysis

Continuous epidural (1.0 mm) and MXene EEG2/EMG recordings were performed in the (author university) Mouse Neurobehavioral Laboratory for 2 d starting 7 d after implantation using Pinnacle amplifiers (sampling 400 Hz). MXene HdEEG recordings and 0.1 mm epidural EEG2 studies were performed for 3 h using a Nicolet V32 amplifier sampled at 250 or 500 Hz 1, 2, and 3 weeks after surgery. Electrode impedances across the skull (MXene) were measured using Nicolet's proprietary protocol; because the outputs of the Pinnacle prefabricated 1.0 mm headmounts were bipolar, the epidural impedances of single 1.0 mm Pinnacle epidural electrodes could not be measured.

To record convulsive tonic–clonic seizures, mice were injected intraperitoneally with pentylenetetrazol (PTZ, Sigma-Aldrich), dissolved in saline (1 mg/ml, 7 mM). The mice were first injected with 40 mg/kg PTZ; if a convulsive seizure did not occur within 10 min, an additional 60 mg/kg was injected.

A reviewer blinded to mouse identity and treatment identified EEG segments for analysis. For the continuous EEG2/EMG recordings, EEG/EMG spectrograms were generated using the EDFbrowser software. Awake epochs were identified as periods of high-power EMG and low-delta (≤4 Hz) EEG power, and epochs of slow wave sleep (SWS) were identified as periods of low-power EMG and high-delta EEG power (Louis et al., 2004). In addition, the logarithmic *δ*/*ϴ* spectral power ratios were calculated in 10 s windows in overlapping 1 s intervals using a fast Fourier transform with a Hanning taper and with *δ* frequencies of 1–4 Hz and *ϴ* frequencies of 5–7 Hz. Awake epochs had lower *δ*/*ϴ* spectral power ratios, and SWS epochs had higher *δ*/*ϴ* spectral power ratios.

To compare the EEG signal quality obtained from the two electrode types, we quantified the movement artifact during 30 min samples of wakefulness. A blinded reviewer experienced in identifying movement artifacts reviewed awake EEG obtained with 1.0 mm epidural (the left hemisphere referenced to the cerebellum) and MXene (M3 electrode −1.85 mm M/L, −0.25 mm A/P referenced to the midbrain) using the EDFbrowser software and marked the beginning and end of movement artifact. The seconds of movement artifact per hour of awake EEG were then calculated.

Five segments (10 s) of awake and SWS EEG without artifact or epileptiform discharges were chosen from each mouse for quantitative analysis. In addition, SWDs were identified as high-voltage rhythmic 6–8 Hz waveforms (Robinson and Gilmore, 1980; Sitnikova and Van Luijtelaar, 2007). Five spike–wave discharge (SWD) segments were chosen from each mouse from 5 s before to 2 s after SWD onset (*t*_0_ defined as the time of the first SWD spike).

EEG analysis was performed using the FieldTrip MATLAB toolbox (Oostenveld et al., 2011). EEG2 and HdEEG were downsampled to a sample frequency (Fs) of 200 Hz and bandpass filtered (BP) between 0.5 and 55 Hz using a two-pass windowed-sinc finite impulse response filter (Widmann et al., 2015). For quantification of HdEEG preictal spectral power and network connectivity and for the comparison of MXene EEG signal on Days 7, 14, and 21, the EEG was also band-stop (BS) filtered at 60 Hz using a two-pass Butterworth notch filter.

As another method of comparing the SWD EEG2 spectral power between MXene and epidural 1.0 mm electrodes and to account for the voltage differences between these electrode types and days postimplantation, we *Z*-transformed the EEG2 traces before performing spectral analysis. We first used the MATLAB “detrend” function to remove the fitted mean from the data and then used the MATLAB “envelope” function to calculate the root mean square of the signal with a sliding window of 10 s. The *Z*-transformed signals were calculated as the detrended signals divided by the root-mean-square envelope.

EEG2 and HdEEG spectral power and HdEEG cross-spectral density were calculated using a two-cycle Morlet transform from 2 to 55 Hz. The EEG2 Morlet transforms were performed every 50 ms from 0.5 to 9.5 s of the awake background/SWS and from 0.5 to 1.5 s of the ictal SWD and then averaged among those timepoints. The spectral power of PTZ-evoked convulsive seizures was calculated using the same Morlet transform every 50 ms of the first 5 s of the seizure. The HdEEG Morlet transforms were performed every 50 ms from 5 s before to 2 s after SWD *t*_0_. The spectral density and cross-spectral density measurements were averaged among the five awake/SWS/SWD segments from each mouse.

To compare SWD preictal increases in β-frequency spectral power recorded with MXene HdEEG with those obtained with previous studies, the preictal (0.10 to 1.00 s prior to *t*_0_) β-spectral frequency was normalized to the preictal baseline (3.00–5.00 s before *t*_0_) as decibels (dB), which are calculated as follows:

$$\mathrm{dB}=10\;X\;log_{10}\left(\frac{P_{\mathrm{preictal}}}{P_{\mathrm{baseline}}}\;\right)$$

where *P*_preictal_ and *P*_baseline_ are the preictal and baseline spectral power, respectively.

We used the weighted phase lag index (WPLI), a network measure that is insensitive to volume conduction (Vinck et al., 2011), to determine the effects of mTBI on SWD preictal network connectivity. The HdEEG preictal Morlet transform cross-spectral density measurements were averaged in overlapping 1 s bins spaced every 0.5 s from 1 to 5 s before SWD onset. The 2–30 Hz WPLI between each electrode pair was then calculated at these time points. A bootstrap method was used to calculate the WPLI 95% confidence interval and the WPLI values with confidence intervals that did not overlap zero were considered suprathreshold (McGuinness et al., 2022). The network node degree, the number of suprathreshold connections, was determined at each electrode.

### Statistical analyses

Statistics were calculated using R for Windows version 4.1.3. The Shapiro–Wilk test was used to determine if variables were normally distributed. Fabrication and surgery times and differences in mTBI/sham righting times were compared using two-tailed *t* tests. Differences between epidural and MXene electrodes in movement artifact times were determined using the Wilcoxon signed-rank test and the median (Md), first and third quartiles (Q1, Q3), and bootstrapped confidence interval (CI) are presented. Perioperative deaths (within 7 d) were compared using the chi-squared test. Differences in the HdEEG preictal channel/frequency/time distribution of spectral density and network node degree were calculated using cluster-based permutation (CBP) analyses. CBP analysis is a nonparametric statistical method (Maris and Oostenveld, 2007) that addresses the multiple comparison problems inherent in analyzing numerous electrode, frequency, and time combinations by clustering nearby electrode, frequency, and times based on either initial repeated measure (comparing preictal and baseline spectral power) or independent (comparing mTBI with sham) *t* tests. The summed *t* score of the clusters was determined. The data sets were then randomly permuted 500 times, and the percentile of the initial summed *t* score versus the permuted *t* scores is the probability that the spatiospectral–temporal distributions differ. Here, the CBP analyses were run with spatial clusters including electrodes within 2 mm and a critical cluster statistic of 0.025; *p* values were appropriately adjusted to account for two-tailed comparisons.

## Results

### Array fabrication and implantation

Top and bottom views of completed MXene HdEEG arrays are shown in Figure 1, *C* and *D*. Despite having 16, rather than 2 channels, the fabrication time of the MXene HdEEG arrays (97 ± 5 min) was significantly shorter than that of the epidural 0.1 mm EEG2 arrays (179 ± 57 min; *p* = 0.004). Impedance spectroscopy demonstrated that the epidural 0.1 mm and MXene electrodes had similar electrode/electrolyte impedances at frequencies between 1 and 1,000 Hz (Fig. 1*E*) with a maximal difference of only 3.1 kΩ at 1 Hz.

Despite having a larger number of channels, the average duration of the implantation surgery for MXene HdEEG electrodes (55 ± 4 min, *N* = 7) was significantly shorter than that for epidural 0.1 mm EEG2 arrays (73 ± 4 min, *N* = 12, *p* < 0.001). The mice tolerated the implantation of all types of arrays. Two of the 31 MXene HdEEG implanted mice died in the perioperative period (within 7 d), and there were no perioperative deaths in the 0.1 mm (*N* = 14) or 1.0 mm (*N* = 6) epidural-implanted mice (*χ*^2^ = 1.343, *p* = 0.51).

### EEG2 recordings with epidural and MXene electrodes

**Figure 2.**
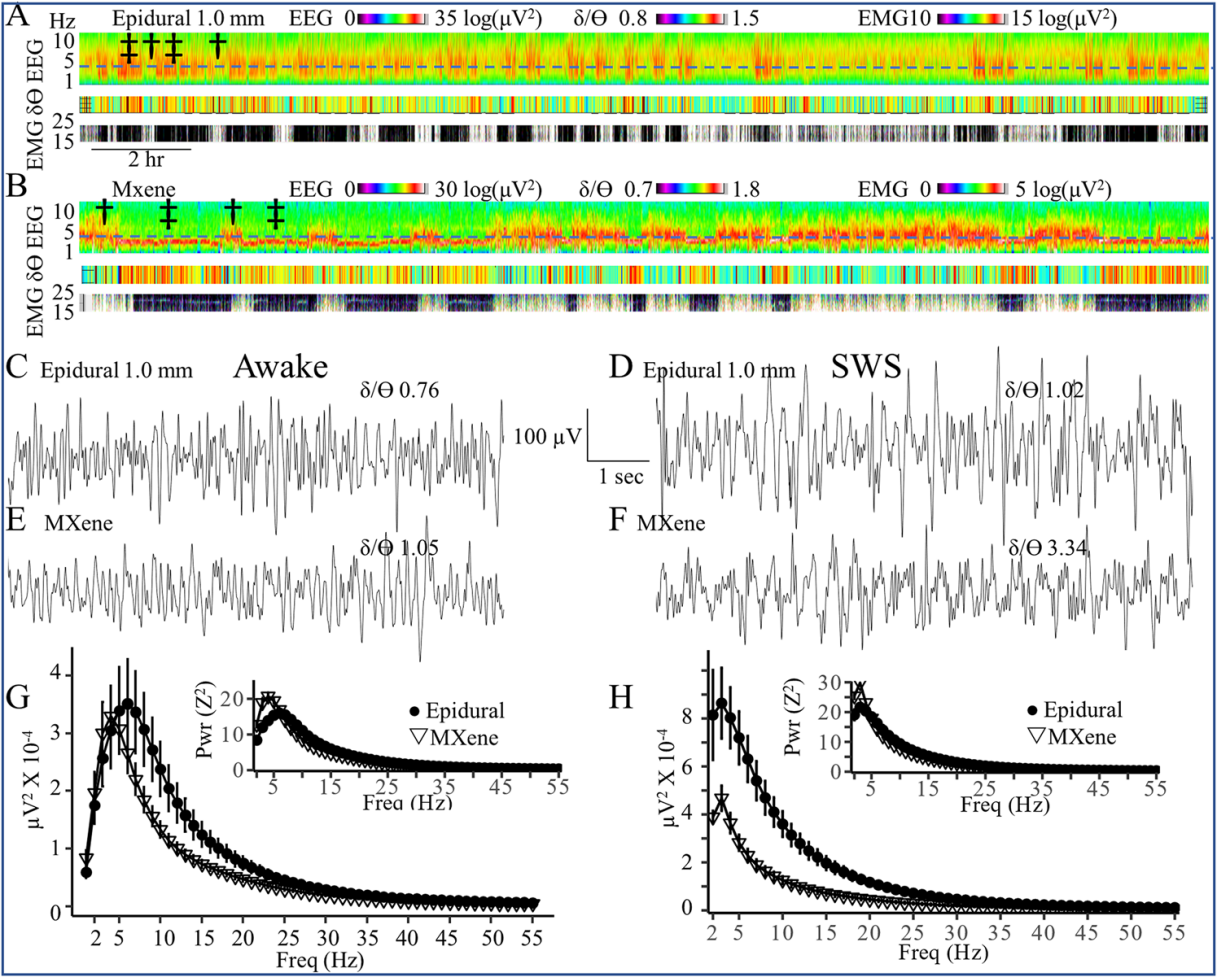
Epidural and MXene EEG2 recordings of wakefulness and SWS. ***A***,***B***, Sample 24 h EEG spectral power, *δ*/*ϴ* power ratio, and EMG spectral density plots from EEG2 recordings obtained with Pinnacle amplifiers 7 d after implantation with 1.0 mm epidural (***A***) and MXene (***B***) electrodes. The dashed blue lines indicate 4 Hz. Time periods with increased EMG power, decreased delta (≤4 Hz) spectral power, and decreased *δ*/*ϴ* ratio indicate wakefulness (examples marked with a single dagger, †). Periods with decreased EMG power, increased delta (≤4 Hz) spectral power, and increased *δ*/*ϴ* ratio indicate SWS (examples marked with a double dagger, ‡). EMG associated with the Pinnacle epidural electrodes and those with the MXene electrodes are on different power scales because Pinnacle EMG wires are uninsulated while the MXene EMG wires are insulated except at the tips. Sample EEG2 recordings obtained 7 d after implantation of wakefulness (***C***,***E***) and SWS (***D***,***F***) with (***C***,***D***) 1.0 mm epidural electrodes (Pinnacle left frontal electrode referenced to the cerebellum) and (***E***,***F***) MXene electrodes (channel M3 −1.85 mm M/L, −0.25 mm A/P referenced to the midbrain, 11 kΩ skull impedance). The *δ*/*ϴ* ratio values for awake and SWS samples are given in each panel. Mean awake (***G***,***H***) SWS spectral power ± SEM obtained with epidural (●, *N* = 6) and MXene (▽, *N* = 5) electrodes. Insets in ***G*** and ***H*** depict the spectral power of *Z*-transformed EEG. Fs = 200 Hz, BP 0.5–55 Hz.

We performed continuous 48 h EEG2 recordings using 1.0 mm epidural and MXene electrodes. EEG and EMG spectral density (Fig. 2*A*,*B*) were analyzed to identify periods of wakefulness (low *δ* power, low *δ*/*ϴ* power ratios, and high EMG power) and SWS sleep (high *δ* power, high *δ*/*ϴ* power ratios, and low EMG power). Differentiation between awake and SWS states was clear with both electrode types. SWS recorded with epidural electrodes had increased *ϴ* as well as *δ* power, and thus the SWS-associated increases in *δ*/*ϴ* power ratios were smaller for epidural electrodes than MXene electrodes.

To compare the signal quality of the two electrode types, we quantified the movement artifact during 30 min samples of wakefulness. There were no significant differences in movement artifacts between the epidural (9, 3–38, 3–272 s/h; Md, Q1–Q3, CI; *N* = 5 mice) and MXene (20, 1–54, 0–74 s/h, Md, Q1–Q3, CI; *N* = 6 mice, *p* = 0.52) electrodes.

Figure 2, *C* and *F*, presents the sample EEG2 traces obtained with epidural (C,D) and MXene (E,F) electrodes in wakefulness (C,E) and SWS (Fig. 2*D*,*F*). Raw spectrograms of awake and SWS EEGs are shown in Figure 2, *G* and *H*, with insets depicting the corresponding spectrograms of *Z*-transformed EEG. The raw spectrogram of awake EEG (Fig. 2*G*) shows that awake recordings by epidural and MXene EEG2 exhibit similar distributions of spectral density although the MXene electrodes (peak 4 Hz, 33,006 ± 5,201 µV^2^) exhibit somewhat decreased power in frequencies above 6 Hz than the epidural electrodes (peak 6 Hz, 35,144 ± 7,619 µV^2^). The larger contribution of lower frequencies during wakefulness in MXene electrodes is apparent in the spectrogram of *Z*-transformed EEG (Fig. 2*G*, inset). Compared with the awake state, the peak frequencies in SWS for both electrode types were lower (3 Hz) and of greater magnitude for both the epidural (86,382 ± 14,766 µV^2^) and MXene (46,760 ± 5,253 µV^2^), although there is a greater relative increase in epidural electrodes (146%) than in MXene electrodes (42%).

**Figure 3.**
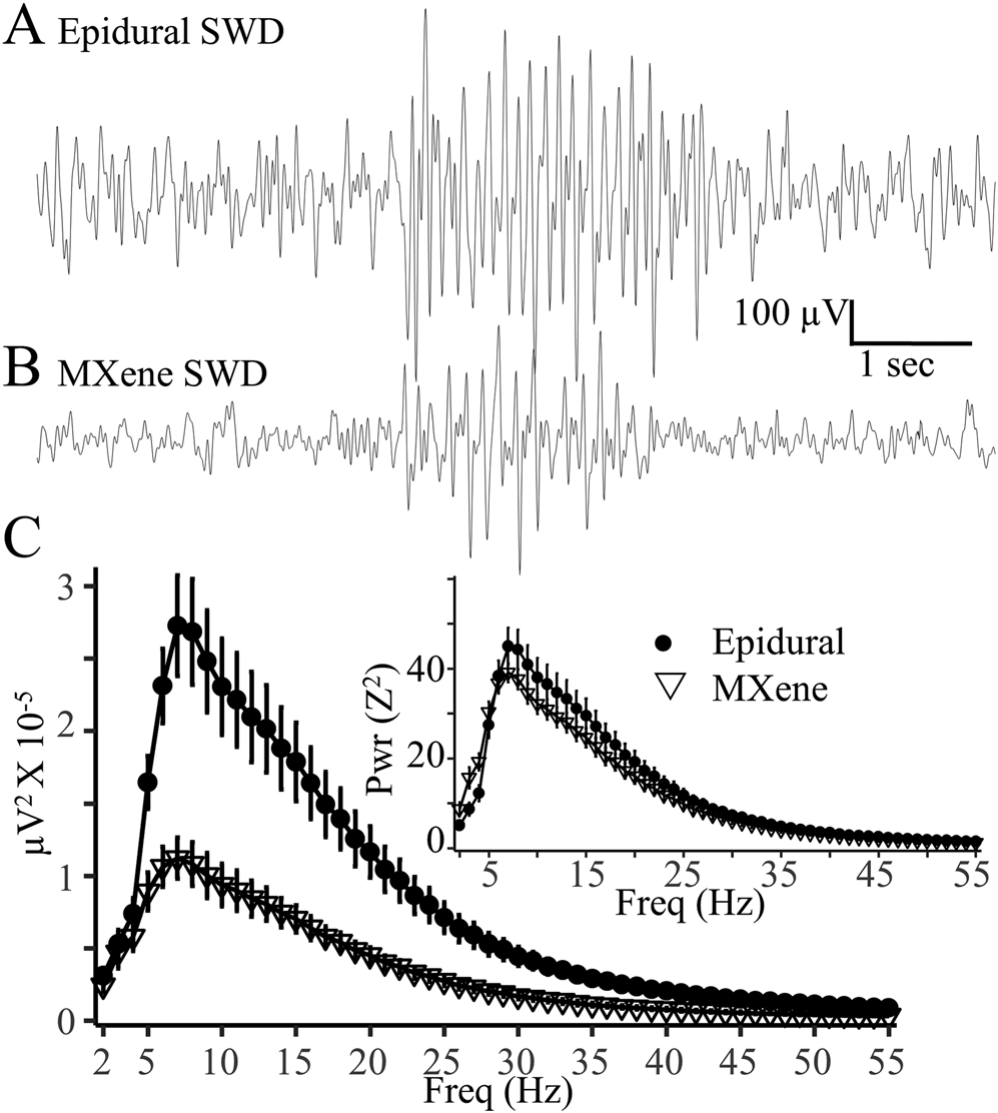
Epidural and MXene EEG2 SWD recordings. ***A***, Sample EEG2 recordings of SWDs obtained with Pinnacle amplifiers 7 d after implantation with (***A***) epidural 1.0 mm electrodes (Pinnacle left frontal electrode referenced to the cerebellum) or (***B***) MXene skull surface electrodes (channel M3 −1.85 mm M/L, −0.25 mm A/P referenced to the midbrain, 19 kΩ skull impedance). Fs = 200 Hz, BP 0.5–55 Hz. ***C***, SWD mean spectral power ± SEM obtained with epidural (●, *N* = 6) and MXene (▽, *N* = 5) electrodes. Inset SWD mean spectral power of *Z*-transformed EEG.

Next, we compared the EEG2 recordings of epileptiform SWDs obtained with 1.0 mm epidural and MXene electrodes during continuous EEG2 recording. SWDs obtained with both types of electrodes showed the typical spike, positive transient, and wave components reported previously in rodent absence seizures (Robinson and Gilmore, 1980; Sitnikova and Van Luijtelaar, 2007). Spectrograms of the SWD spectral density are shown in Figure 3*C*. The epidural 1.0 mm electrodes had a greater amplitude of raw spectral power than the MXene electrodes but both electrode types exhibited a broad peak of spectral density from 6 to 30 Hz reflecting the 6–8 Hz primary SWD frequency along with the first, second, and third harmonics. The inset in Figure 3*C* is the average SWD spectrogram with EEGs that were first *Z*-transformed and demonstrates that although SWDs have a higher voltage when recorded with epidural than MXene electrodes, they have the same distribution of spectral density.

No 1.0 mm epidural electrodes or MXene electrodes were lost during the 2 d continuous video-EEG monitoring recording. To compare the durability of MXene electrodes with 0.1 mm epidural electrodes, those that can be used for epidural HdEEG recordings, we made weekly 3 h EEG2 recordings of MXene (*N* = 11) and 0.1 mm epidural electrodes (*N* = 11) 1, 2, and 3 weeks after implantation. While 82% of the epidural 0.1 mm preparations lost at least one electrode within 3 weeks of surgery, only 18% of the MXene implants lost at least one electrode at 3 weeks.

**Figure 4.**
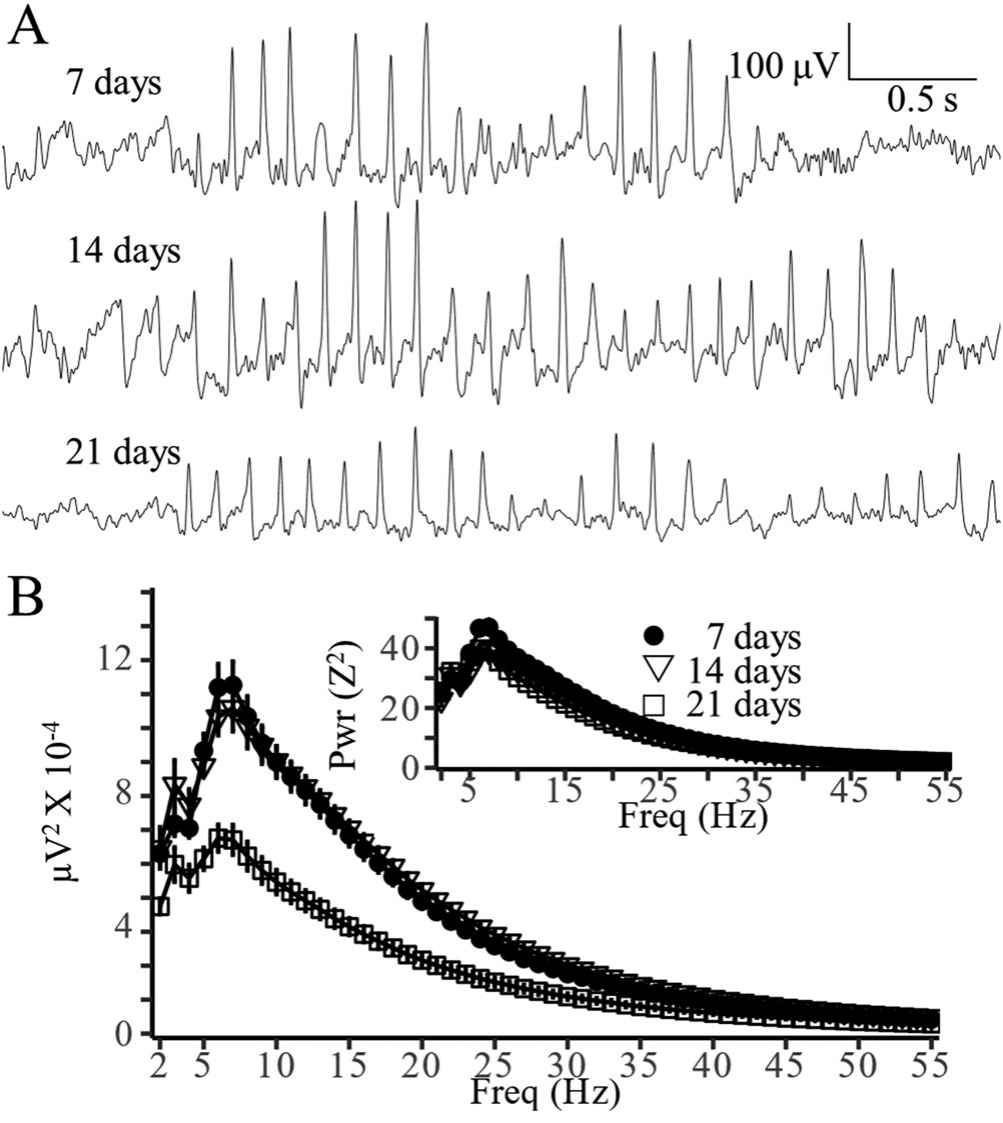
Stability of MXene EEG2 recordings. ***A***, Sample MXene EEG2 recordings of SWDs obtained with a Nicolet amplifier from the same mouse 7 (19 kΩ skull impedance) and 14 (19 kΩ), and 21 (25 kΩ) days after electrode implantation (channel M3 −1.85 mm M/L, −0.25 mm A/P referenced to the midbrain Nicolet amplifier, Fs = 200 Hz, BP 0.5–55 Hz, BS 60 Hz). ***B***, SWD mean spectral power ± SEM obtained at 7 (●, *N* = 6), 14 (▽, *N* = 5), 21 (□, *N* = 6) days after implantation. Inset: SWD means spectral power ± SEM of Z-transformed EEG.

Figure 4*A* depicts MXene electrode SWD recordings obtained from the same mouse 7, 14, and 21 d after electrode implantation. The SWDs in all three recordings are similar although the voltage of the one obtained at 21 d is somewhat lower than on Days 7 and 14. The mean spectral power tracings of SWDs obtained from mice at 7 d (*N* = 6), 14 d (*N* = 5) and 21 d (*N* = 6) are shown in Figure 4*B*. The distribution of SWD spectral power is similar at each timepoint although the spectral power at Day 21 is approximately 30% lower at all frequencies compared with the mice recorded on Days 7 and 14. The inset of Figure 4*B* depicts the mean spectral power of *Z*-transformed EEG recordings of SWDs and demonstrates similar spectral profiles at all three timepoints, a result that indicates that although the voltage of the 21 d recordings is less than that at other timepoints, the ratio of the SWD signal to background EEG variability remains unchanged.

**Figure 5.**
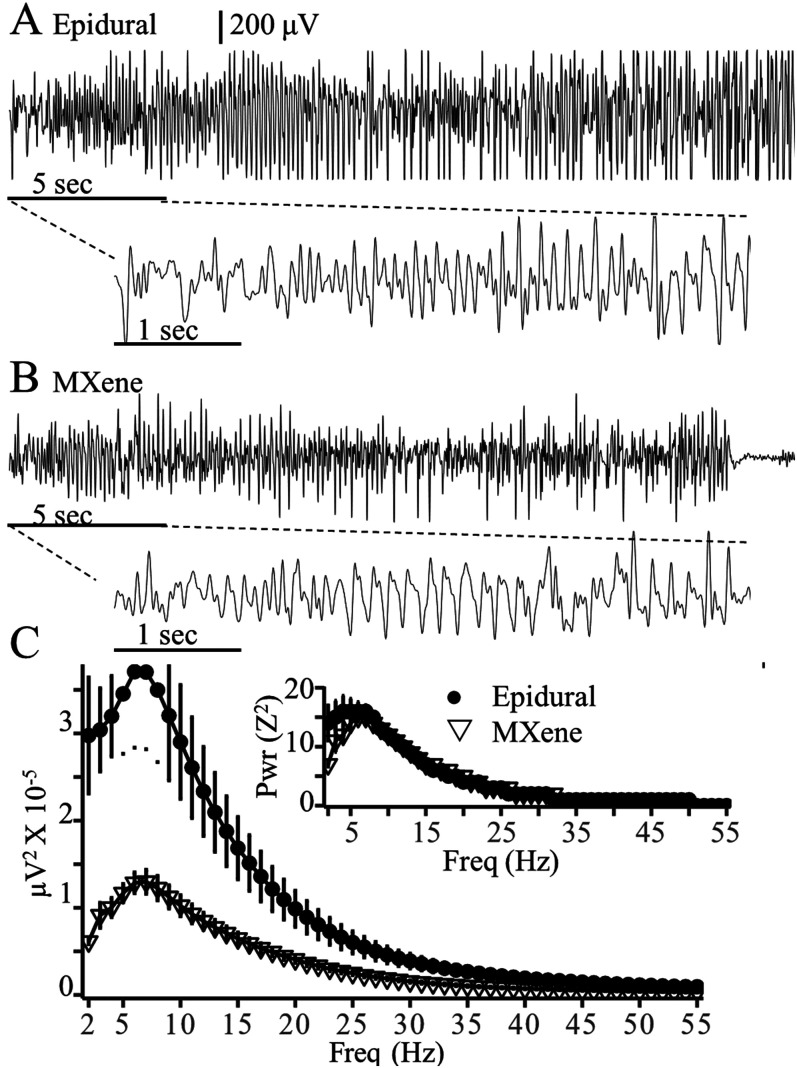
Epidural and MXene EEG2 recordings of tonic–clonic seizures Sample PTZ-evoked tonic–clonic seizures recorded with Pinnacle amplifiers 7 d after implantation with (***A***) 1.0 mm epidural (Pinnacle left frontal electrode referenced to the cerebellum) or (***B***) MXene (channel M3 −1.85 mm M/L, −0.25 mm A/P referenced to the midbrain, 10 kΩ skull impedance) electrodes. The bottom traces in ***A*** and ***B*** depict the first 5 s of the seizures on an expanded timescale. ***C***, Mean ± SE spectral power of the first 5 s of tonic–clonic seizures obtained with epidural (●, *N* = 5 mice) and MXene (▽, *N* = 5 mice) electrodes and the inset is the spectral power of the *Z*-transformed EEG. Fs 200 Hz, BP 0.5–55 Hz.

MXene and 1.0 epidural electrodes were also durable during convulsive seizures; neither electrode type was lost during the convulsions. Tracings of PTZ-evoked tonic–clonic seizures were similar when obtained with epidural (Fig. 5*A*) and MXene (Fig. 5*B*) electrodes. The lower tracings in Figure 5, *A* and *B*, are the first 5 s of the convulsive seizures depicted on an expanded time scale to demonstrate that convulsive movements do not prevent the identification of EEG epileptiform waveforms. Figure 5*C* demonstrates the mean spectral power of the first 5 s of PTZ-evoked convulsive seizures recorded with epidural (*N* = 5 mice) and MXene (*N* = 5 mice) electrodes, and the inset is the spectral power of the *Z*-transformed EEG. The spectral profiles of PTZ-evoked convulsive seizure onset were similar when recorded 1.0 mm epidural and MXene electrodes; however, as expected, the absolute spectral power was lower for the MXene electrodes (peak 7 Hz, 1,29,917 ± 13,730 μV^2^) than 1.0 mm epidural electrodes (peak 6 Hz 3,70,898 ± 87,240 μV^2^).

### MXene 16 channel HdEEG SWD recording

We next recorded 16-channel HdEEG using the MXene electrodes. Like the M3/M4 electrodes, the additional MXene HdEEG electrodes, with the exception of S5p and S6p, provided excellent EEG signals for at least 3 weeks (data not shown). However, the S5p and S6p electrodes in the lateral posterior region were more likely than the other HdEEG to become dislodged early after implantation (Week 1, with probabilities of 0.38 and 0.31, respectively).

**Figure 6.**
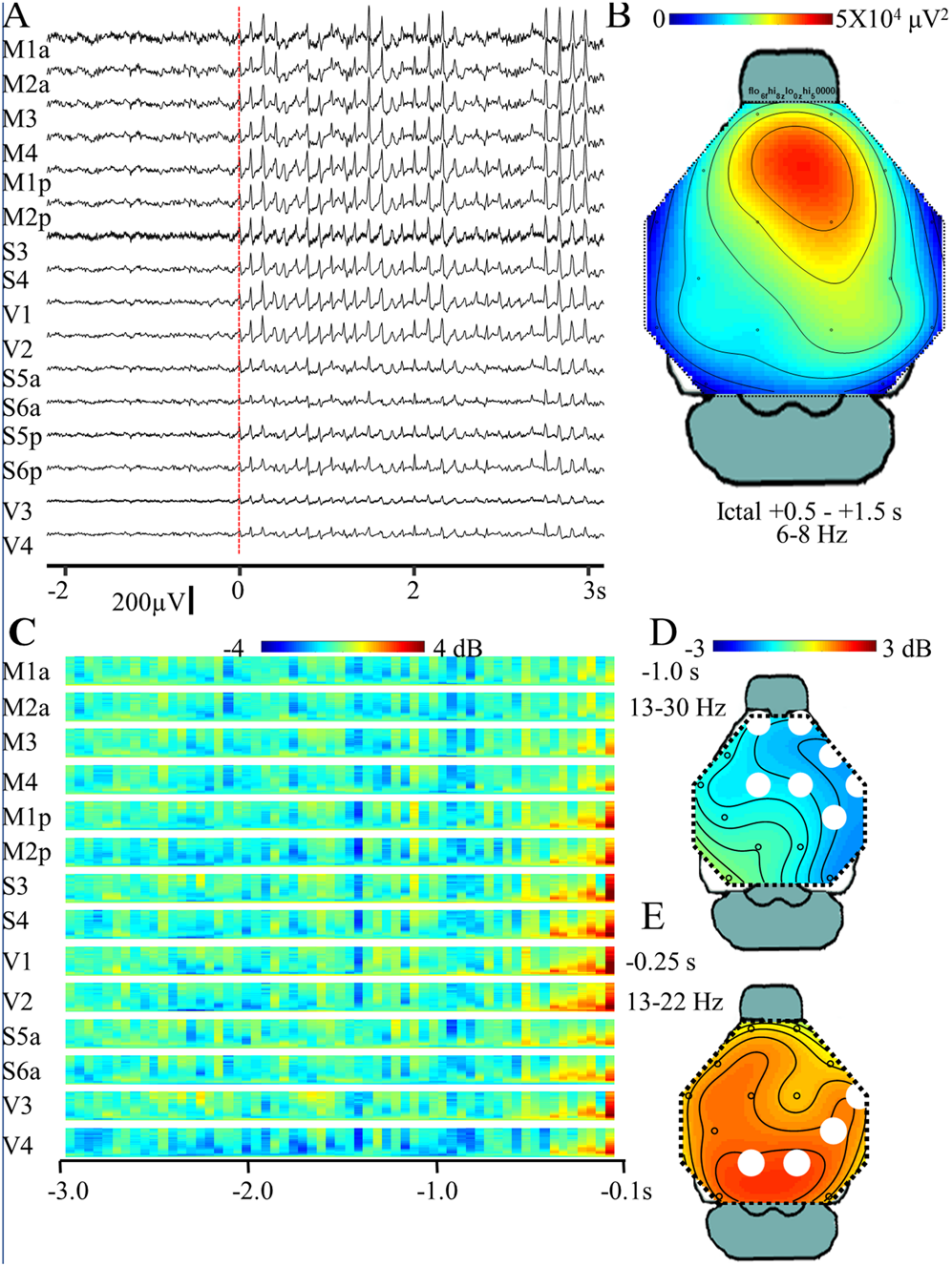
MXene HdEEG SWD. ***A***, Sample HdEEG recording of a SWD obtained with a Nicolet amplifier 7 d after implantation with MXene skull surface HdEEG. SWD onset (*t*_0_, marked with a red line) is the time of the first spike. Fs = 200 Hz, BP 0.5–55 Hz, BS 60 Hz, mean impedance 29 ± 2 kΩ. ***B***, SWD mean ictal spectral density (6–8 Hz, 0.5–1.5 s) topology plot (*N* = 12). ***C***, Mean preictal β-frequency (13–30 Hz, each *y*-axis) baseline-normalized spectral power at each HdEEG channel plotted from 3.0 to 0.1 s prior to SWD onset. Times −1.00 to −0.10 s statistically compared with awake background 13–30 Hz power (*N* = 12, *p* = 0.006). ***D***,***E***, Topology plots of mean normalized spectral density at (***D***) 1.00 s (13–30 Hz) and (***E***) 0.25 s (13–22 Hz) prior to SWD onset. The white circles indicate electrode clusters found in the CBP analysis.

Previous studies used epidural HdEEG electrodes to map the spatial distribution of SWDs (Ding et al., 2019), and thus we recorded SWDs with MXene HdEEG electrodes and compared the SWD spatial distribution with those of the prior reports. A HdEEG SWD tracing is depicted in Figure 6*A*, and a topology plot of the mean 6–8 Hz spectral power of the SWD onset (0.5–1.5 s after the first spike) is shown in Figure 6*B*. As seen previously with epidural HdEEG electrodes (Ding et al., 2019), the characteristic 6–8 Hz spikes and waves are recorded in all electrodes over the dorsal cortex, but with prominence in the frontal electrodes over the motor cortex.

Prior experiments with epidural electrodes also found small, but significant, preictal increases in β-frequency spectral density just prior to SWD onset (Sorokin et al., 2016; Ding et al., 2019), and thus we determined if these findings could be observed with MXene HdEEG electrodes. Figure 6*C* depicts the mean preictal (3.00–0.10 s before *t*_0_) β-frequency (13–30 Hz) normalized spectral density and demonstrates an increase in β-frequency spectral power starting at approximately 0.40 s prior to SWD onset, a finding consistent with previous results. Interestingly, there was also a brief decrease in spectral power in the frontocentral electrodes at approximately 1.00 s before the SWDs. CBP found a significant difference (*p* = 0.006) in β-spectral power between the baseline (3–5 s prior to SWD) and the preictal period (0.10–1.00 prior to SWD). Prominent frequency/time point CBP clusters included the decreased 13–30 Hz spectral power in the frontocentral electrodes at −1.00 s (Fig. 6*D*) and increased 13–22 Hz power in the posterior electrodes at −0.25 s (Fig. 6*E*).

### Effect of mTBI on preictal network connectivity

A family history of epilepsy is a significant risk factor for the development of seizures after mTBI (Christensen et al., 2009). Possibly, mTBI exacerbates mild preexisting epileptic network. Since MXene HdEEG can record SWDs without intracranial burr holes that can potentially confound mTBI experiments, we used MXene HdEEG to determine the effects of mTBI on the SWD preictal network. One week after MXene HdEEG implantation, mice underwent a 2 h baseline recording followed by either mTBI or sham. The mTBI produced no difference in visible behavior or righting times (mTBI 107 ± 19 s, sham 113 ± 17 s). Subsequent HdEEG recordings at 1 d and 7 d after mTBI/sham demonstrated that mTBI did not disrupt MXene electrode recording quality. Postmortem examination of the brains found no visible evidence of injury in either group (data not shown).

**Figure 7.**
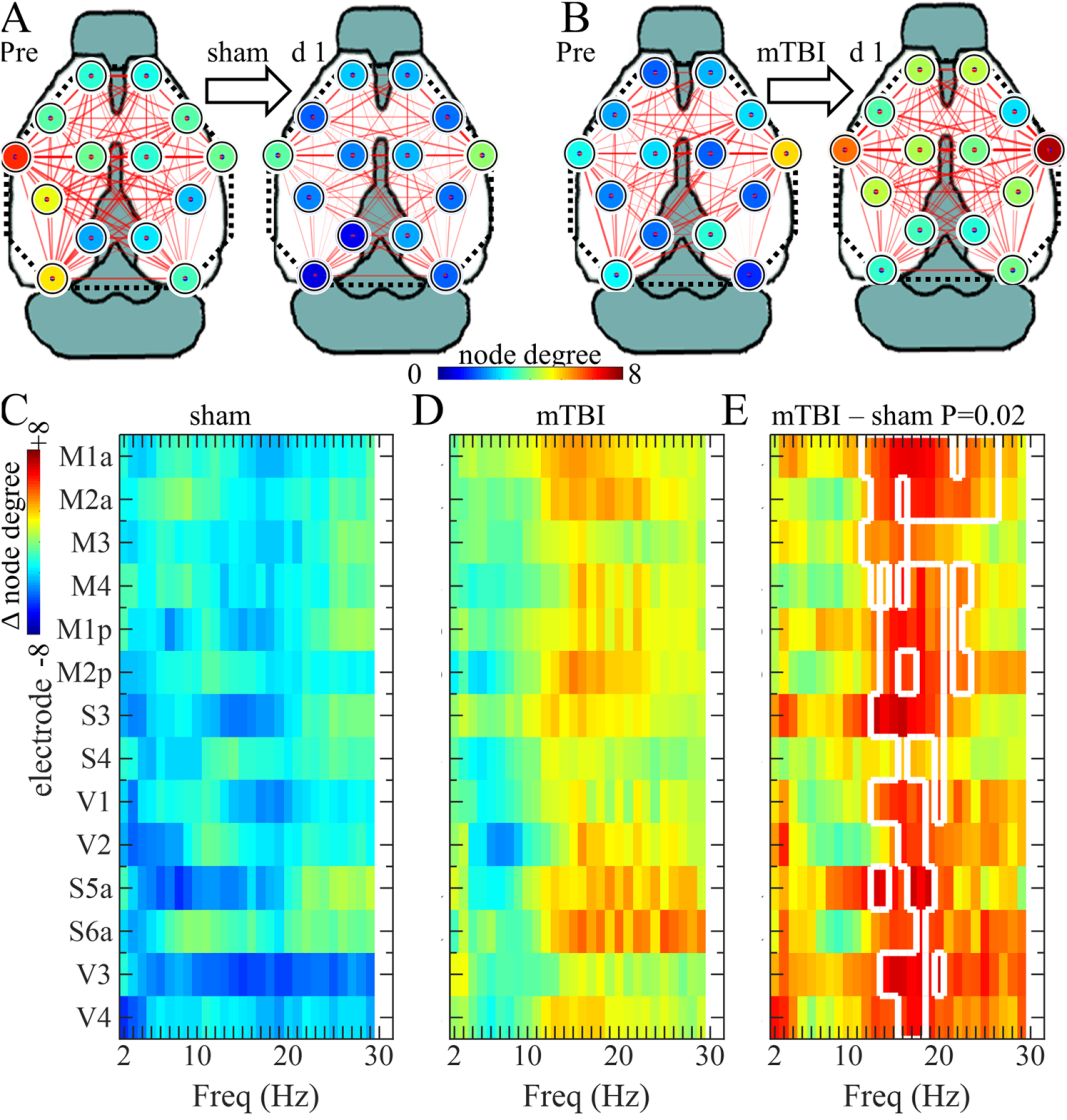
Effects of mTBI on preictal SWD network connectivity. ***A***,***B***, Mean 13 Hz network node degree (colored circles at electrode positions) and WPLI connectivity (red lines between the electrodes, line intensity corresponds to WPLI values) at 1.5 s before SWD for mice that underwent (***A***) sham exposure (*N* = 7 mice) or (***B***) mTBI (*N* = 8 mice). The mean WPLI/degree prior to sham/mTBI is depicted on the left of the panel and those after sham/mTBI are on the right. Grids depict mean differences in 2–30 Hz network node degree between (***C***) post and prior sham, (***D***) post and prior mTBI, and (***E***) mTBI and sham at all channels at 1.5 s prior to SWD onset. CBP statistically tested differences between sham and mTBI in network node degree changes (*p* = 0.02) with clusters including most channels at times 1.0 (14–20 Hz) and 1.5 (13–20 Hz) seconds prior to mTBI. The white lines enclose channel/frequency clusters at 1.5 s prior to SWD onset.

To determine the effects of mTBI on SWD preictal network connectivity, we calculated the interelectrode WPLI and network node degree at frequencies 2–30 Hz from 1 to 5 s prior to SWD onset. While sham mice exhibited a decreased preictal network connectivity from the pre-sham recording to 1 d post-sham (Fig. 7*A*,*C*), mTBI increased preictal network connectivity (Fig. 7*D*). We used CBP to statistically compare sham and mTBI on changes in network node degree from 2 to 30 Hz and at times from 1 to 5 s prior to ictal onset (Fig. 7*E*, *p* = 0.02). Clusters in CBP testing were at 1.0 and 1.5 s prior to SWD onset and included β-frequencies (13–20 Hz) in almost all channels. Although there were also increases in network node degree on Day 7, these changes were not statistically significant (*p* = 0.06, data not shown).

## Discussion

This study demonstrated high-quality skull surface EEG2 and HdEEG recordings using open-source MXene-based EEG arrays. The signals recorded with MXene electrodes were comparable to those obtained with epidural electrodes, albeit with an expected lower voltage and preferential attenuation of faster frequencies. MXene HdEEG recordings revealed that mTBI produced acute changes in preictal SWD connectivity, despite the absence of visible behavioral differences. These results demonstrate that the MXene skull surface electrode array is a novel, inexpensive tool that allows a minimally invasive mechanism to study mouse neurophysiology.

### Comparison of MXene electrodes with current techniques

Compared with current epidural EEG2 and HdEEG methods (Wasilczuk et al., 2016; Ding et al., 2019), this open-source method of MXene electrode skull surface EEG eliminates the need for drilling skull burr holes. This advance represents a major refinement in animal care. Eliminating skull burr holes also mitigates potential confounding factors in rodent physiology experiments, especially those designed to study the effects of TBI or those investigating the physiology of young mice. MXene EEG array fabrication is more efficient than the epidural open-source techniques given that its layout and wiring are achieved using PCBs and its electrode pads are easily coated with MXene. Importantly, surgical implantation of MXene arrays is easier and more efficient than epidural electrodes since burr holes are not required and may thus be performed by laboratory personnel with less experience than those who typically perform intracranial surgeries.

Nanofabricated 16- and 32-channel skull surface HdEEG arrays are commercially available (NeuroNexus) and are substantially thinner (15 µm) than the ones produced by our open-source method. However, although the electrodes of the commercial arrays lie on the skull surface, chronic placement of this array still requires three skull-penetrating anchoring screws (Jonak et al., 2018) and thus is problematic for mTBI studies and experiments using young mice with thin skulls. Another substantial advantage of our open-source MXene HdEEG system compared with commercially available arrays is reduced cost (US$6.64 vs US$778). Although a technique has been reported allowing retrieval and reuse of the commercial HdEEG up to six times (Jonak et al., 2018), the cost per use of the commercial array is still substantially greater than that of the MXene HdEEG system. Therefore, our open-source method will be especially beneficial for investigators needing to screen many subjects with chronic EEG.

### mTBI acutely increases preictal network connectivity

Although previous studies used epidural EEG2/limited-channel EEG to measure the effects of mTBI in mice (Lim et al., 2013; Modarres et al., 2017; Ichkova et al., 2020; MacMullin et al., 2020), this is the first report, to our knowledge, that measured the effects of mTBI on EEG using non-penetrating electrodes or with HdEEG. We found that although mTBI did not change righting time compared with sham exposure and there were no visible behavior differences between mTBI and sham mice, mTBI did acutely increase SWD preictal network connectivity with the most notable increases in β-spectral frequency. Mild TBI increases the risk of developing epilepsy (Annegers et al., 1998; Christensen et al., 2009; Karlander et al., 2021), and early seizures, those that occur within 7 d of the injury, may increase that risk (Lowenstein, 2009). Because epileptiform SWDs are preceded by increased β-spectral power (Ding et al., 2019; Sorokin et al., 2016, and Fig. 6) increased network connectivity in the β-frequency band (Fig. 7) may raise the risk of early seizures. Future studies will determine the association of mTBI, β-spectral power, and early seizures.

### Study limitations

Intermittent recordings demonstrated that although SWD waveform morphology and relative spectral power distributions were the same on Day 21 as on Days 7 and 14, the voltage was reduced, possibly due to increased electrode skull impedance (Fig. 4). Longer studies will need to be performed to determine the maximum duration electrodes provide quality recordings. Moreover, the lifespan experiment (Fig. 4) was performed using intermittent weekly 3 h recordings. Possibly, the MXene electrode lifespan would be reduced even further during continuous recordings with electrode wires causing continuous tension on the surface array. Therefore, future long-duration recordings should be performed continuously and/or with a wireless acquisition system.

This study demonstrated that MXene electrode recordings successfully localized the 6–8 Hz epileptiform discharges to the anterior region (Fig. 6*B*), captured low-voltage, high-frequency preictal β-activity (Fig. 6*C*,*D*), and identified the increased mTBI-associated β-frequency network connectivity (Fig. 7). However, it should be emphasized that epileptiform activity is of higher voltage than physiological rhythms and that the β-frequency activity (Figs. 6, 7) was both generalized and time-locked to SWD onset. Our study did not determine if MXene skull surface electrodes would be sufficient to localize focal low-voltage physiological rhythms as might be obtained in mouse behavioral experiments. In fact, we found that compared with epidural recordings, MXene EEG attenuated theta/alpha frequencies during wakefulness (Fig. 2). Additional studies will need to be performed to test the ability of MXene skull surface EEG to detect low-voltage electrophysiological activity that would otherwise be identified with intracranial electrodes.

### Conclusions

In conclusion, this study determined that open-source MXene EEG2 and HdEEG electrodes represent an inexpensive, convenient method for recording mouse EEG sleep/wake cycles, epileptiform activity, and preictal rhythms/connectivity without skull disruption. This represents a refinement in animal care. Using MXene HdEEG recordings, we determined that mTBI increased preictal SWD network connectivity, a change that could increase SWDs early after mTBI. Future studies will determine the long-term longevity of MXene skull surface electrodes in wired and wireless acquisition systems and the capability of these electrodes to detect low-voltage electrophysiological activity typically identified with intracranial electrodes.

## Synthesis

Reviewing Editor: Viji Santhakumar, University of California Riverside Decisions are customarily a result of the Reviewing Editor and the peer reviewers coming together and discussing their recommendations until a consensus is reached. When revisions are invited, a fact-based synthesis statement explaining their decision and outlining what is needed to prepare a revision will be listed below. The following reviewer(s) agreed to reveal their identity: Aristea Galanopoulou.

The authors have provided several revisions to the study in response to the summary comments. However, experimental substantiation of certain key aspects are still not fully adequate. It is suggested that the authors provide relevant data and stats to support certain aspects that need further validation or be explicit about the limitations.

The response to the concern about the comparison between HdEEG and standard screw electrode during movement artifacts is inadequate. The authors need to show data (EEG traces) to compare the quality of recordings during movements. Only short baseline segments (Fig 2) and seizures (one with each type of electrodes, single channel) are shown in figure 4 which is condensed at a temporal scale that does not allow such discrimination. Longer duration data segments comparing the HdEEG electrode performance to screw electrodes during seizures and movements is needed. Additionally, detailed annotations/explanation on type of acquisition system, reading montage and filters, behavior of the mice during the recordings would strengthen the manuscript. These should be readily available in the existing data sets.

Important details about the new electrode need to be better represented in the figures. Such documentation should include impedance values (on the skull if possible), longevity of electrodes, electrode position and signal quality. For example, Figure 3C implies that the signal amplitude is lower in the HdEEG. However, it is difficult to draw a conclusion without knowing where the electrodes are located. Additionally, it would be useful to show/compare the signal HdEEG electrode signal over time within the same animal to demonstrate signal stability over time. While these studies are reported anecdotally, the representative traces and data need to be part of the manuscript.

Figure 5: 0 point is not set at the onset of the SWD; data need to be revised by correcting the 0 point. Appropriate determination of SWD onset is critical for the data presented here.

Authors should provide a more thorough assessment of the study limitations based on the tests conducted in this study. Continuous EEG recordings were limited to 48h: this should be clearly stated. Intermittent recordings for few hours a week up to 3 weeks do not provide confidence that the electrodes will stay in place during continuous recordings for weeks (line 305). Also, discussion of stability of electrodes should be based on data in the manuscript.

It would be useful to provide examples of the sleep and awake patterns using the HdEEG recordings; the authors must have such data if they have done 48hr recordings.

Figure 2 recording is still ambiguous. There is a shift of the MXene trace to delta which is unconvincing for awake. In any case, for electrophysiologists it would be important for an HdEEG array to show whether there is good anteroposterior organization during sleep wake and this is not shown here. Part of the reason this is important is because the placement of electrodes over the skull would render the signal weaker and the artifacts and noise stronger and signal frequencies would be possibly altered compared to an epidural electrode. Thus, what work for large amplitude SWDs may not be as effective in physiological sleep-wake rhythms.

Inspection of skulls and brains post-mortem is not sufficient to exclude underlying pathology due to excessive pressure trying to create a burr hole with the needle. Was nay staining for inflammatory markers or glia performed?

## Author Response

November 10, 2023

Dear eNeuro editors,

We are pleased to submit the revised version of our manuscript, "An Open Source Mouse Chronic EEG Array System with High Density MXene-Based Skull

Surface Electrodes" (eN-OTM-0512-22). We appreciate the positive comments

from the reviewers and acknowledge the reviewers' time and effort in

providing helpful suggestions to improve the manuscript.

We addressed all the reviewer's comments in detail below and marked new

text in the manuscript with highlighting.

Detailed Responses to Reviewer's Questions

1. Reviewer: Since the intended novelty of the authors' work is the

technology, it would be better to improve the examples of wake-sleep

recordings which currently are not doing justice to EEGs one would expect

to get at this age of rodents or delete as the ones offered are not

satisfactory.

1. Response: We provided improved examples of awake and sleep recordings

in Figure 2. We also indicated the delta/theta power ratio for each of

the examples (see further explanation of this metric in response 2). In

these examples, SWS is associated with higher amplitude of lower

frequency (delta band <= 4Hz) power. As discussed in response 2, SWS

recorded with Pinnacle epidural electrodes is associated with increased

delta and theta band power while SWS recorded with MXene electrodes is

associated with increased delta, but not theta band power. Therefore,

while SWS recorded with both electrodes is associated with increased

delta/theta power ratio, there is a greater increase in the delta/theta

ratio for MXene electrodes (1.05 to 3.34) than for Pinnacle epidural

electrodes (0.76 to 1.02).

2. Reviewer: Figure 2: Clarify what A and B show. In panel B the bursts

of activity in the EEG correspond to EMG bursts, suggesting these could

be artifacts. But in panel A, bursts of EMG correspond to attenuated EEG

activity which is contra-intuitive; unless synchronization is wrong

or other quality issues exist. Thus, A and B cannot demonstrate that the

two systems see the same activity.

2. Response: We added three features in Figure 2A-B to assist the readers

in identifying SWS. Wakefulness is defined as low-amplitude, mixed

frequency EEG with increased EMG activity while SWS is high-amplitude,

low frequency (i.e. delta band <= 4Hz) power and without REM associated sawtooth waves (Louis et al, 2004). These SWS criteria are

present in distinct states for both the epidural (Pinnacle, A) and MXene

(B) recordings. Therefore, as a first method assist readers in

identifying SWS, we placed a dashed line at 4 Hz of the spectrograms. For

both Pinnacle (A) and MXene (B) electrodes there are clear states of

increased delta power below the 4 Hz dashed line that correspond to EMG

attenuation - these periods are SWS.

2

There is a difference between SWS recorded with epidural (Pinnacle,

A) and MXene electrodes. SWS recorded with epidural Pinnacle electrodes

is also associated with increased theta

band (5-7 Hz) power in addition to increased delta band power. It is this

SWS-associated increased delta and theta power that likely prompted the

reviewer to comment, "in panel A, bursts of EMG correspond to attenuated

EEG activity which is contra-intuitive." Therefore, as a second method

to assist readers in rapidly identifying SWS, we calculated the ratio of

delta to theta power and plotted this delta/theta ratio metric between

the EEG and EMG spectrograms. For both Pinnacle epidural (A)

and MXene (B) electrode recordings, SWS is associated with increased

delta power (below 4 Hz line), decreased EMG power, and increased

delta/theta ratio. As expected, given the increased SWS-associated theta

power with the Pinnacle electrodes, the increase in delta/theta ratio for

the Pinnacle electrodes is smaller than that for the MXene electrodes.

However, the delta/theta ratio is increased for both systems.

Finally, as a third method to assist the reader in identifying SWS,

we placed several single dagger (†) markers for examples of wakefulness

and double dagger (‡) markers for examples of SWS. At single dagger (†)

points of wakefulness the reader can clearly see little/no delta power

below the 4 Hz line, increased EMG power, and reduced delta/theta ratio

while at the double dagger (‡) markers of SWS the reader can clearly see

increased delta power below the 4 Hz line, decreased EMG power, and

increased delta/theta ratio.

3. Reviewer: In panels C-F, the EEG differences are not as clear as

expected between awake and SWS, with the exception of D. If better

examples exist it would be preferred.

3. Response: As discussed in response 1, we provide new examples of

awake/sleep.

4. Reviewer: Figure 4: If EEG was read using band pass filters 0.5 - 55,

what is the point of band-stop filter at 60Hz? What type of filters were

used?

4. Response: The bandpass filter was a two-pass windowed-sinc finite

impulse response filter and the band-stop 60 Hz filter is a two pass

Butterworth filter. We added the term "two-pass" to the description of

the windowed-sinc FIR BP filter to the methods and added that the band stop filter is a two pass Butterworth filter. For the Nicolet amplifier,

we used a band-stop filter in addition to the 0.5-55 Hz because the roll

off characteristics of the FIR BP filter still allowed some 60 Hz

artifact to be present (i.e. the BP filter is not a hard-stop filter). As

discussed in the rebuttal letter of the last revision, the Nicolet

amplifier does not have a zero-gain preamplifier at the mouse head and

thus some 60 Hz artifact is intermittently present and thus a band-stop

filter was applied consistently to all Nicolet-acquired data. Please note

that the amplifier type is separate from the new technology being

described here (MXene surface electrodes) which can be used with any type

of amplifier. MXene electrodes are used successfully in this study with

Pinnacle amplifiers that possess headstage preamplifiers and do not

require notch filtering.

3

5. Reviewer: Figure 6: How does correcting 0 point not affect the results

which remain exactly the same?

5 Response: The calculations in Figure 6 (and for all SWDs) were made

using the time of first spike as time zero (see Methods). This was done

by mathematically finding the first negative peak and this did not change

between submissions. HdEEG tracing figures (e.g. 6A) were made using a

ggplot2 script from the extracted MATLAB EEG data and did not change

between submissions. However, to keep consistent font styles and

formatting among figures, the ggplot2 graphic results were then labeled

using a vector drawing program. In a previous submission, the labeling of

time zero was inadvertently shifted was then corrected in a later

submission. Thus, it was only a change with the drawing program for

labeling and not the data.

6. Reviewer: Abstract: "Moreover, increased preictal beta frequency

network connectivity may to the development of early post-mTBI seizures":

the study looked at SWD not mTBI seizures. The authors need to change the

statement.

6 Response: We changed the abstract to state post-mTBI SWDs.

We thank the reviewer and editors again for their time and effort and for

the helpful comments that have improved this manuscript. We hope it is

now suitable for publication in eNeuro.

Sincerely,

The corresponding author
